# A prospective Phase III trial evaluating patient self‐reported pain and cosmesis in accelerated partial breast irradiation utilizing 3‐D versus intensity‐modulated radiotherapy

**DOI:** 10.1002/cam4.4242

**Published:** 2021-09-01

**Authors:** Charles E. Leonard, Yunfei Wang, Lina Asmar, Rachel Y. Lei, Kathryn T. Howell, Phyllis L. Henkenberns, Timothy K. Johnson, Tracy L. Hobart, Shannon P. Tole, Jane M. Kercher, Jodi L. Widner, Lora Barke, Terese Kaske, Dennis L. Carter

**Affiliations:** ^1^ Rocky Mountain Cancer Centers Littleton Colorado USA; ^2^ Linasmar Consulting Houston Texas USA; ^3^ SurgOne Greenwood Village Colorado USA; ^4^ Sally Jobe Diagnostic Breast Center Greenwood Village Colorado USA

**Keywords:** accelerated partial breast, breast cancer, radiotherapy

## Abstract

**Purpose/Objective:**

The primary objective is to examine patient self‐assessment of breast pain and cosmesis between three‐dimensional (3D‐CRT) versus intensity‐modulated radiotherapy (IMRT). The secondary objective is to evaluate any relationship of treatment planning conformality of both cohorts to patient‐assessed pain. Assessments were performed at interim 12, 24, 36, and 48 months with a final 5‐year assessment.

**Materials/Methods:**

In total, 656 patients (3D‐CRT *n* = 328; IMRT *n* = 328) were randomly assigned to either IMRT or 3D‐CRT accelerated partial breast radiotherapy to 38.5 Gy in 10 BID 3.85 Gy fractions.

**Results:**

Median follow‐up was 3 years. Multivariate analysis showed that pain severity significantly decreased from baseline to the 12‐month follow‐up visit (<0.001 for both 3D‐CRT and IMRT) in each cohort. There was significantly less pain at 2 (*p* = 0.002) and 3 years (0.045) in the IMRT arm versus the 3D‐CRT arm when compared to the baseline pain level. There was no difference in patient‐assessed cosmesis at any follow‐up point; however, although MD‐assessed cosmesis showed no difference from years 1 to 4, there was significantly better cosmesis for 3D‐CRT versus IMRT (*p* = 0.047) at 5 years. There was a significant correlation between a maximum pain score and an increase in the CI_100_ (indicating less conformity) in the IMRT cohort (*p* < 0.01) and in the IMRT subgroup when the CI_100_ was ≤0.37 cohort arm (*p* = 0.01).

**Conclusion:**

In the analysis of our primary objective we found that at 2 years, IMRT resulted in more interval improvement in breast pain after baseline when compared to patients treated with 3D‐CRT planning. As seen in our secondary analysis, this may be due to the ability of IMRT to achieve higher conformality (as evidenced by lower CI values) resulting in less fibrosis. There were no differences in patient‐assessed cosmesis or MD‐assessed cosmesis for years 1–4; however, physician‐assessed 5‐year cosmesis was better with 3D‐CRT.

## Graphical abstract

After a median follow‐up of 3 years, results showed that the IMRT cohort experienced significantly less pain at 2 and 3 years. Patients did not describe any differences in breast cosmesis; however, physicians did score 3D‐CRT with significantly better cosmesis at 5 years (only 147 patients) but no differences in years 1–4.

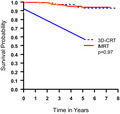

## 1 INTRODUCTION

Standard of care for early stage invasive breast cancer includes breast‐conserving lumpectomy and whole‐breast radiotherapy (BCT). This treatment combination results in survival statistics which are comparable to mastectomy.^1^, ^2^, ^3^, ^4^, ^5^ The current standard of care, whole‐breast irradiation (WBI) over 3–4 weeks, can be a deterrent for patients who would otherwise be good candidates for BCT. Due to socioeconomic factors, 5–6 weeks of whole‐breast radiotherapy can be an impediment for some patients eligible for breast conservation.^6^, ^7^, ^8^ Recently there has been increasing evidence to support a shortened course of partial breast radiotherapy, accelerated partial breast radiotherapy (APBI), mostly utilizing three‐dimensional radiotherapy.^9^, ^10^, ^11^, ^12^, ^13^, ^14^, ^15^, ^16^, ^17^, ^18^, ^19^, ^20^, ^21^, ^22^, ^23^, ^24^, ^25^, ^26^, ^27^

Intensity‐modulated radiation therapy (IMRT) is a planning/treatment modality which may have a higher integral dose but it allows a higher degree of conformality to the target and facilitates lower doses to critical structures resulting in a lower toxicity profile without a loss of efficacy. This has been shown in several disease sites.^28^, ^29^, ^30^, ^31^, ^32^, ^33^, ^34^, ^35^, ^36^, ^37^

A primary goal of our prospective Phase III study was to evaluate if there is any difference in pain or breast cosmetic profiles between two cohorts of patients treated with either 3D‐CRT or IMRT APBI. This report compares the dosimetric data and clinical outcomes of APBI delivered with three‐dimensional conformal radiation therapy (3D‐CRT) and IMRT. Preparatory phase II investigations of APBI utilizing IMRT have supported the hypotheses that IMRT resulted in increased conformality, reduced volume of normal tissue receiving high doses, and reduced chest wall/lung/heart doses. Furthermore, achievable improvements in conformality and reduced doses to the chest wall have been associated with a reduction in patient reports of pain.^24^, ^25^, ^26^, ^27^ Therefore, the authors have engaged in conducting a randomized trial to detect any differences in breast pain or cosmesis between 3D‐CRT APBI and IMRT APBI cohorts.

## 2 MATERIALS AND METHODS

### 2.1 Patient population

In total, 656 patients with pathological stage *T*_0_, *T*_1_, and *T*_2_ (<3 cm) N0 breast cancer, total disease span (invasive with DCIS or DCIS) less than 3 cm, and a minimum of 2 mm negative margins were prospectively enrolled on an Institutional Review Board‐approved randomized APBI IMRT versus 3D‐CRT. Eligibility requirements for study enrollment included ≥40 years of age, ≤3 cm focus maximum diameter of invasive/intraductal carcinoma, and ≥2 mm margins. Patients with ER and/or PR‐negative or HER 2/neu‐positive tumors were not excluded. Patients were randomized by bias coin flip methodology. Patients with gross multifocal disease were excluded. One hundred and ten patients were excluded in this analysis. One hundred and six patients were excluded for lack of follow‐up (death‐5; lost to follow‐up‐12; missing visits‐83; moved‐5; withdrew consent‐1) and four for MD decision (cosmetic surgery‐1; mastectomy‐1; recurrence‐2). Prescription dose was 3.85 Gy delivered twice daily to the partial breast over five consecutive days for a cumulative dose of 38.5 Gy. Target volumes included surgical bed, plus an additional 10 mm margin for the clinical target volume (CTV), and an additional 5 mm margin for planning target volume (PTV). The CTV and PTV excluded the skin (external 5 mm). Additional treatment techniques including dosimetry target volume dose requirements and normal tissue constraints have been previously published in detail.^24^, ^25^, ^26^, ^27^

### 2.2 3D‐CRT treatment planning

A supine treatment planning CT scan with a thickness of ≤0.5 cm was used. Wedge techniques or manual forward planned “field within a field” techniques were allowed to improve dosimetric coverage. Customized blocking was utilized to encompass the PTV with an additional 0.5 cm margin for penumbra. A minimum of four 3‐D fields were utilized.

### 2.3 IMRT treatment planning

A supine treatment planning CT scan with a thickness of ≤0.3 cm was used. Either a sliding window or step and shoot technique were acceptable. A minimum of 95% of the prescribed dose was delivered to 95% of the CTV. A minimum of 95% of the PTV received more than 95% of the prescribed dose of 38.5 Gy.

### 2.4 Conformality indices

Conformality indices for the 100%, 75%, and 50% isodose lines using the PTV as the target volume were calculated by the following formula:

$$\text{CI}=\left[\left(\text{PIV}‐\text{TVPIV}\right)/\text{PIV}\right]‐\left[\left(\text{TVPIV}‐\text{TV}\right)/\text{TV}\right]$$

Where CI is the conformity index, PIV is the prescription isodose volume (i.e., 100%, 75%, or 50%), TVPIV is the volume of the target volume (i.e., PTV, CTV, and gross target volume [GTV]) receiving a dose equal to or greater than the PIV, and TV is the target volume.

### 2.5 Follow‐up schedule

Follow‐up visit intervals were calculated from radiotherapy (RT) completion. Follow‐up visits occurred at the following intervals: 1 month, 4, 8, 12, 16, 20, and 24 months, then yearly through year 5 (and beyond year 5 with patient permission).

Mammography was performed between 3 and 6 months after radiotherapy completion as a new post‐surgery and RT baseline. The number and position of fiducial markers, if used, were noted.

Case report forms (CRFs) were to be completed at each study follow‐up visit. CRFs completed by investigators at the time of follow‐up were considered source documents. Adverse event reporting and cosmetic grading were required at each visit using the Radiation Therapy Oncology Group [RTOG] breast cosmesis scale. Patient‐reported outcomes at each visit included an analysis of self‐reported pain and cosmesis.

For all subjects that consented to breast photography, digital images were to be taken at baseline and 12, 36, and 60 months after RT completion for future blinded comparison.

### 2.6 Statistics

Mean with standard deviation and median with interquartile ranges (IQRs) were presented for continuous variables and numbers with percentages were tabulated for categorical variables. Two sample means and proportions were compared using the Student's *t*‐test and the Pearson chi‐squared test or Fisher's exact test, respectively. To account for repeated patient's clinic visits with every 12 months, the generalized estimating equations (GEEs) approach using the multinomial distribution with the cumulative logit link was applied to the estimate odds ratios of the two study treatments associated with pain and MD‐assessed cosmesis. Age was adjusted in models. Survival analyses were evaluated by the Kaplan–Meier estimator with a log‐rank test used for comparison between groups. SAS version 9.4 (SAS Institute Inc.) was used for all data analysis. A sample size of 660, equally divided between IMRT and 3D‐CRT, affords 85% power to detect a one‐sided difference (with overall α of 0.05) of 0.23 standard deviations between treatment arms (assuming up to 15% of subjects will be unevaluable due to incomplete assessments).

All subjects who met eligibility were subjected to randomization. No stratifying factors were used in the protocol. Those subjects who were declined medical benefits for IMRT for reasons independent of their technical eligibility for either arm crossed over to the 3D‐CRT arm. Study documents were updated to document the reason for cross over. If treatment arms became unequal, block randomization was utilized to maintain simultaneous accrual to both arms. Each block of 30 treatment assignments was generated randomly using biased coin randomization to include 15 or more IMRT assignments with the remaining assigned to 3D‐CRT. Whenever the study arms contain unequal number of subjects, the randomization within each block will favor the deficient arm.

## 3 RESULTS

### 3.1 Patient population

Between July 2009 and April 2015, 656 patients were enrolled. Median follow‐up was 3 years for both the IMRT and 3D‐CRT cohorts. Patient characteristics are shown in Table 1. Table 1 shows that most patients (3D‐CRT [97%] and IMRT [98%]) were treated using fiducial‐based image‐guided radiotherapy (IGRT), and rarely cone beam IGRT. Patients were treated at five Rocky Mountain Cancer Center facilities in the Denver, Colorado metropolitan area.

**TABLE 1:** Patient demographic and baseline disease characteristics

Parameter	3D‐CRT *n* = 328	IMRT *n* = 328	*p* value
Age (in years)			
Mean (SD)	63.1 (10.2)	61.0 (9.6)	0.006
Median (Q1, Q3)	64.3 (56.3, 70.0)	61.6 (53.8, 66.9)	
Race [*n* (%)]			
Caucasian/White	309 (94.2)	310 (94.5)	0.329
Asian	2 (0.6)	4 (1.2)	
American Indian or Alaska Native	3 (0.9)	0	
Black or African American	4 (1.2)	2 (0.6)	
Native Hawaiian or other Pacific Islander	1 (0.3)	0	
Hispanic/Latino	9 (2.7)	10 (3.1)	
Other/Unknown	0	2 (0.6)	
Tumor size [mean (SD)]	1.08 (0.61)	1.09 (0.56)	0.719
Margin size [mean (SD)]	0.69 (0.40)	0.69 (0.34)	0.735
Histology [*n* (%)]			
IDCA	232 (70.7)	260 (79.3)	0.034
ILCA	23 (7.0)	21 (6.4)	
DCIS	71 (21.7)	45 (13.7)	
Other invasive	0	1 (0.3)	
IMC (invasive mammary carcinoma)	2 (0.6)	1 (0.3)	
ER status [*n* (%)]			
Positive	302 (92.1)	310 (94.5)	0.244*
Negative	23 (7.0)	16 (4.9)	
Unknown––insufficient tissue	2 (0.6)	2 (0.6)	
Missing	1 (0.3)	0	
HER2 NEU status [*n* (%)]			
Positive	18 (5.5)	19 (5.8)	0.957*
Negative	240 (73.2)	258 (78.7)	
Not performed	68 (20.7)	47 (14.3)	
Unknown––insufficient Tissue	2 (0.6)	3 (0.9)	
Missing	0	1 (0.3)	

Summary: There is a statistically significant difference of age between two arms (*p* = 0.006). The mean (SD) is 63.1 (10.2) for 3D‐CRT and 61.0 (9.6) for IMRT.

* *p* value between negative and positive.

### 3.2 Patient‐assessed pain

**FIGURE 1:**
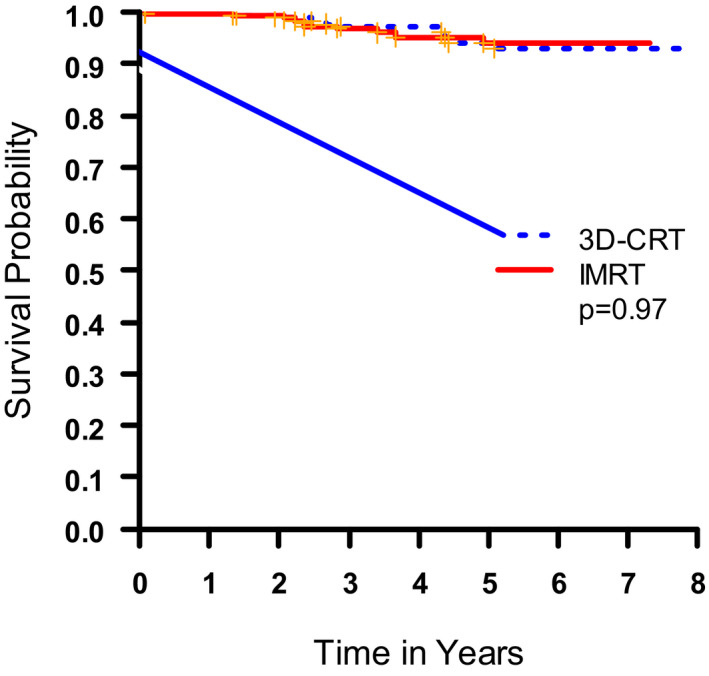
Kaplan–Meier estimation of overall survival

Table 2 and Figure 1 show a summary of patient‐assessed pain by follow‐up visit which illustrates that there were no significant differences between the 3D‐CRT and IMRT cohorts at each follow‐up. Table 2 shows that 64.4% and 66.7% versus 53.9% and 60.3% of patients had no pain in the IMRT and 3D‐CRT cohorts, respectively, at 24‐month and 36‐month follow‐up. Table 3 illustrates not only that there is significantly decreasing pain for the age of the patient but also that when the visit was used as a categorical variable and models were adjusted by age, the probability of a decreasing change of pain score when *compared to baseline*, was statistically significantly better at each follow‐up visit for all patients (both cohorts) at all visits. Table 4 also shows that the IMRT cohort experienced significantly more resolution of breast pain than the 3D‐CRT cohort at the 24‐month and 36‐month follow‐up (*p* = 0.002 and 0.045, respectively).

**TABLE 2:** Summary of pain grade by visits

Time	Pain	3D‐CRT	IMRT	*p* value
At baseline [*n* (%)]	No pain	116 (42.2)	101 (37.3)	0.059
	Mild tenderness or infrequent discomfort	137 (49.8)	134 (49.4)	
	Mild frequent pain, not generally interfering with daily activities	21 (7.6)	28 (10.3)	
	Moderate and constant pain, interfering with daily activities	0	6 (2.2)	
	For example, severe pain, requiring narcotics	1 (0.4)	2 (0.7)	
	Total	275	271	
At 12 month [*n* (%)]	No pain	131 (52.8)	133 (55.0)	0.317
	Mild tenderness or infrequent discomfort	98 (39.5)	89 (36.8)	
	Mild frequent pain, not generally interfering with daily activities	16 (6.5)	15 (6.2)	
	Moderate and constant pain, interfering with daily activities	1 (0.4)	5 (2.1)	
	For example, severe pain, requiring narcotics	2 (0.8)	0	
	Total	248	242	
At 24 month [*n* (%)]	No pain	96 (53.9)	113 (64.6)	0.116
	Mild tenderness or infrequent discomfort	73 (41.0)	57 (32.6)	
	Mild frequent pain, not generally interfering with daily activities	9 (5.1)	5 (2.9)	
	Total	178	175	
At 36 month [*n* (%)]	No pain	73 (60.3)	84 (66.7)	0.584
	Mild tenderness or infrequent discomfort	41 (33.9)	37 (29.4)	
	Mild frequent pain, not generally interfering with daily activities	7 (5.8)	5 (4.0)	
	Total	121	126	
At 48 month [*n* (%)]	No pain	46 (51.7)	48 (55.2)	0.742
	Mild tenderness or infrequent discomfort	41 (46.1)	36 (41.4)	
	Mild frequent pain, not generally interfering with daily activities	2 (2.2)	3 (3.4)	
	Total	89	87	
At 60 month [*n* (%)]	No pain	52 (72.2)	44 (58.7)	0.177
	Mild tenderness or infrequent discomfort	18 (25.0)	24 (32.0)	
	Mild frequent pain, not generally interfering with daily activities	2 (2.8)	6 (8.0)	
	For example, Severe pain, requiring narcotics	0	1 (1.3)	
	Total	72	75	

**TABLE 3:** Results of multivariate analysis for pain score using GEE by study arm and visits

Parameter	Odds ratio	95% CI	*p* value
Model 1			
3D‐CRT arm			
Age	0.98	0.96–0.99	0.010
Visit, baseline	Ref.		
12 month	0.68	0.50–0.92	0.011
24 month	0.63	0.45–0.88	0.007
36 month	0.50	0.33–0.75	<0.001
48 month	0.62	0.40–0.96	0.031
60 month	0.28	0.17–0.47	<0.001
Model 2			
IMRT arm			
Age	0.97	0.95–0.99	<0.001
Visit, baseline	Ref.		
12 month	0.49	0.36–0.66	<0.001
24 month	0.30	0.22–0.43	<0.001
36 month	0.29	0.19–0.43	<0.001
48 month	0.43	0.27–0.66	<0.001
60 month	0.41	0.24–0.69	<0.001

**TABLE 4:** Results of multivariate analysis for pain score using GEE between arms and visits

Parameter	Odds ratio	95% CI	*p* value
Age	0.97	0.96–0.98	<0.001
Study arm (IMRT vs. 3D‐CRT)	1.25	0.91–1.73	0.167
Visit, baseline	Ref.		
12 month	0.68	0.51–0.92	0.012
24 month	0.63	0.45–0.88	0.007
36 month	0.50	0.34–0.76	0.001
48 month	0.62	0.40–0.96	0.031
60 month	0.28	0.17–0.47	<0.001
Study arm*visit			
IMRT versus 3D‐CRT at baseline	Ref.		
IMRT versus 3D‐CRT at 12 month	0.71	0.46–1.08	0.107
IMRT versus 3D‐CRT at 24 month	0.47	0.29–0.76	0.002
IMRT versus 3D‐CRT at 36 month	0.56	0.32–0.99	0.045
IMRT versus 3D‐CRT at 48 month	0.68	0.37–1.27	0.227
IMRT versus 3D‐CRT at 60 month	1.45	0.69–3.02	0.327

### 3.3 Physician‐ and patient‐assessed cosmesis

Univariate analysis of the physician‐assessed cosmesis at each follow‐up visit did not differ between modalities (Table 5). At 5 years, 41.7% (3D‐CRT) and 34.7% (IMRT) of patient experienced no/minimal change (0.192). The 12‐month and 24‐month follow‐up visits resulted in a significantly improved cosmesis over baseline for both the IMRT and 3D‐CRT cohorts (Table 6). At 36 months, there was a significant improvement for the 3D‐CRT cohort and a trend for the IMRT cohort. At 60 months, there was a trend for improved cosmesis over baseline only in the 3D‐CRT cohort. Table 7 also illustrates that the only significant difference between the arms was at 5 years when the 3D‐CRT cohort was significantly better than the IMRT cohort (*p* = 0.042) by multivariate analysis. However, follow‐up at this 5‐year time frame is limited with only 75 and 72 patients in the IMRT and 3D‐CRT cohorts, respectively.

**TABLE 5:** Summary of cosmesis appearance grade by visits

Time	Cosmesis appearance	3D‐CRT *n* (%)	IMRT *n* (%)	*p* value
At baseline [*n* (%)]	No change or minimal change	81 (29.6)	84 (31.1)	0.924
	Slightly different	107 (39.1)	108 (40.0)	
	Obvious difference	77 (28.1)	69 (25.6)	
	Drastically different	9 (3.3)	9 (3.3)	
	Total	274	270	
At 12 month [*n* (%)]	No change or minimal change	109 (44.7)	103 (43.3)	0.464
	Slightly different	87 (35.7)	99 (41.6)	
	Obvious difference	43 (17.6)	32 (13.4)	
	Drastically different	5 (2.0)	4 (1.7)	
	Total	244	238	
At 24 month [*n* (%)]	No change or minimal change	72 (40.4)	80 (45.7)	0.364
	Slightly different	74 (41.6)	57 (32.6)	
	Obvious difference	30 (16.9)	36 (20.6)	
	Drastically different	2 (1.1)	2 (1.1)	
	Total	178	175	
At 36 month [*n* (%)]	No change or minimal change	54 (44.6)	49 (38.9)	0.788
	Slightly different	41 (33.9)	50 (39.7)	
	Obvious difference	24 (19.8)	25 (19.8)	
	Drastically different	2 (1.7)	2 (1.6)	
	Total	121	126	
At 48 month [*n* (%)]	No change or minimal change	31 (34.8)	30 (34.5)	0.760
	Slightly different	35 (39.3)	31 (35.6)	
	Obvious difference	22 (24.7)	26 (29.9)	
	Drastically different	1 (1.1)	0	
	Total	89	87	
At 60 month [*n* (%)]	No change or minimal change	30 (41.7)	26 (34.7)	0.192
	Slightly different	24 (33.3)	18 (24.0)	
	Obvious difference	17 (23.6)	29 (38.7)	
	Drastically different	1 (1.4)	2 (2.7)	
	Total	72	75	

**TABLE 6:** Results of multivariate analysis for cosmesis appearance score using GEE by study arm and visits

Parameter	Odds ratio	95% CI	*p* value
Model 1			
3D‐CRT arm			
Age	1.00	0.98–1.02	0.841
Visit, baseline	Ref.		
12 month	0.52	0.40–0.68	<0.001
24 month	0.57	0.42–0.76	<0.001
36 month	0.54	0.38–0.78	<0.001
48 month	0.78	0.52–1.17	0.223
60 month	0.64	0.40–1.01	0.058
Model 2			
IMRT arm			
Age	0.98	0.96–0.99	0.011
Visit, baseline	Ref.		
12 month	0.55	0.42–0.73	<0.001
24 month	0.58	0.42–0.79	<0.001
36 month	0.70	0.49–1.00	0.052
48 month	0.92	0.60–1.40	0.686
60 month	1.21	0.76–1.93	0.433

**TABLE 7:** Results of multivariate analysis for cosmesis appearance score using GEE between arms by visits

Parameter	Odds ratio	95% CI	*p* value
Age	0.99	0.98–1.00	0.0990
Study arm (IMRT vs. 3D‐CRT)	0.89	0.65–1.21	0.447
Visit, baseline	Ref.		
12 month	0.52	0.40–0.68	<0.001
24 month	0.57	0.42–0.76	<0.001
36 month	0.54	0.38–0.78	<0.001
48 month	0.77	0.51–1.15	0.198
60 month	0.63	0.40–1.01	0.055
Study arm*visit			
IMRT versus 3D‐CRT at Baseline	Ref.		
IMRT versus 3D‐CRT at 12 month	1.05	0.72–1.53	0.785
IMRT versus 3D‐CRT at 24 month	1.02	0.67–1.56	0.924
IMRT versus 3D‐CRT at 36 month	1.29	0.78–2.13	0.329
IMRT versus 3D‐CRT at 48 month	1.22	0.68–2.18	0.513
IMRT versus 3D‐CRT at 60 month	1.95	1.01–3.77	0.047

There were no differences in patient‐assessed cosmesis between the two cohorts at any follow‐up point.

### 3.4 Toxicities

There were 6 (1.8%) and 13 (3.9%) grade 3/4 toxicities in the 3D‐CRT and IMRT arms, respectively (*p* = 0.11). Toxicities did not vary significantly between treatment modalities. The main toxicities were skin irritation, followed by fatigue and hyperpigmentation. There were a total of three documented rib fractures. One and two of the fractures were in the IMRT and 3D‐CRT groups, respectively.

### 3.5 Local control and overall and disease‐free survival

There were seven local recurrences in each treatment arm (2.1%, respectively), for a local control probability of 97.9%. The overall survival rates at 2 and 5 years were 99% and 94%, respectively, without differences between the treatment arms. The 2‐year and 5‐year disease‐free survival rates were 98%/97% and 89%/88%, respectively, without differences between 3D‐CRT and IMRT arms.

### 3.6 Conformality indices (CI_100_)

Table 8 shows that conformality indices (CI_100_) as calculated for the study were significantly correlated with the treatment arm. In the IMRT cohort, CI_100_ values (0.31 and 0.29 mean and median values) were significantly lower than 3D‐CRT CI_100_ values (0.44, respectively, for mean and median values) which implies a higher degree of conformality with IMRT treatment planning than 3D‐CRT planning (*p* < 0.001). Table 9 shows that although CI_100_ values correlated with the maximum/baseline pain ratio score in all patients, this effect was primarily observed in the IMRT cohort. Highly conformal CI_100_ values of ≤0.37 correlated with a maximum/baseline ratio score in the IMRT cohort of patients but not in the 3D‐CRT cohort (Table 10). These observations suggest that as CI increases (in increasingly less conformal treatment plans) so does the maximum amount of pain when compared to baseline. However, Table 11 shows that there was no significant correlation between CI_100_ values >0.37 and the maximum/baseline pain ratio score in patients of the IMRT cohort (*p* = 0.08) or patients of the 3D‐CRT cohort (*p* = 0.47).

**TABLE 8:** Mean and median of conformity index (CI100) by study arm

3D‐CRT			IMRT			*p* value
*n*	Mean (SD)	Median (IRQ)	*n*	Mean (SD)	Median (IRQ)	
327	0.44 (0.09)	0.44 (0.38, 0.49)	326	0.31 (0.14)	0.29 (0.22, 0.36)	<0.001

**TABLE 9:** Correlation coefficient between ratio of maximum/baseline pain score and conformity index

		Spearman correlation coefficient (Non‐parametric)	
Parameter	No. of subject	*r_s_*	*p* value
3D‐CRT	325	0.06	0.24
IMRT	326	0.15	<0.01
Total	651	0.10	<0.01

**TABLE 10:** Correlation Coefficient between ratio of maximum/baseline pain score and conformity index (CI100≤0.37)

		Spearman correlation coefficient (Non‐parametric)	
Parameter	No. of subject	*r_s_*	*p* value
3D‐CRT	71	−0.04	0.77
IMRT	259	0.15	0.01
Total	330	0.12	0.02

**TABLE 11:** Correlation coefficient between ratio of maximum/baseline pain score and conformity index (CI100 > 0.37)

		Spearman correlation coefficient (Non‐parametric)	
Parameter	No. of subject	*r_s_*	*p* value
3D‐CRT	254	0.05	0.47
IMRT	67	−0.21	0.08
Total	321	−0.01	0.85

## 4 DISCUSSION

The results of our prospective trial showed an improved pain profile at 2 and 3 years in those patients who were planned and treated with IMRT rather than three‐dimensional planning. Patient‐reported outcomes of cosmesis did not appear to differ between IMRT and 3D‐CRT. However, patient‐reported cosmesis showed no differences and MD‐assessed cosmesis had more equivocal.

There have been few reports detailing the use of IMRT in accelerated partial breast radiotherapy. Jagsi et al. reported unacceptable cosmetic outcomes in their cohort of 32 reported patients.^22^ A comparison of patient characteristics between their study and our report reveals substantial differences in patient planning details especially planning volumes. Our mean planning target volumes were 132.7 and 129.8 cm^3^, respectively, for the 3D‐CRT and IMRT cohorts. Planning volumes in the Jagsi et al. study were 185.5 cm^3^, which is nearly a 30% larger planning volume compared to this study. Our mean planning volume/total breast volumes ratios in the 3D‐CRT and IMRT cohorts of this study were 12% and 11.5%, respectively. Other planning dose constraints specified in the Materials and Methods section of the 2010 article were similar to those of RTOG‐B39. However, our current Phase III partial breast radiotherapy trial did specify eligibility criteria requiring that enrollees have planning volume/total breast volume ratios which are <25%. Mean ipsilateral breast volumes receiving 100% (V100) and 50% (V50) of the prescribed dose were 27.2% and 47.9%, respectively, in the Jagsi et al. report. In their dose‐volume histogram they found that there were significant differences in the breast reference volumes (drawn as breast volume included in a conventional tangent field) between patients with acceptable and unacceptable cosmesis (34.6% vs. 46.1% for V50 of the ipsilateral breast reference and 15.5% vs. 23% for V100 of the ipsilateral breast reference; *p* = 0.02 for both). This suggests that cosmesis may have been more a function of planning volumes than treatment modality. A salient point of these findings would be the importance of planning target volumes, especially as it relates to the PTV and the ipsilateral breast volume. Our mean ipsilateral breast V50 isodose values were 27.1% and 31.4% for the IMRT and 3D‐CRT cohorts, respectively. These values were substantially lower than the values of the Jagsi et al. paper. In addition, margins added to the GTV to construct our target volumes were smaller. Jagsi et al. added margins of 1.5 and 1 cm (total of 2.5 cm), respectively, for the CTV and PTV. In contrast, our respective margins were a total 1 cm smaller at 1 and 0.5 cm (total of 1.5 cm), respectively, for the CTV and GTV.

If one considers whether the poor outcomes reported in the Michigan study could be related to larger planning volumes and greater isodose breast volumes, then it is interesting that two other studies of accelerated partial breast IMRT had very acceptable outcomes when these volumes were smaller. Livi et al.^23^ reported acceptable outcomes when an IMRT APBI cohort was compared to a tangential three‐dimensional whole‐breast cohort in a randomized study. Lewin et al.^21^ observed a 94% excellent/good cosmesis in their IMRT APBI cohort. Interestingly, both studies included either planning and/or isodose volumes comparable to this report.

However, outcomes have not been consistently favorable for three‐dimensional radiotherapy treatment planning for accelerated partial breast treatment as well. Five prospective trials have evaluated the use of 3‐D technology for accelerated partial breast radiotherapy, and only two have concluded that outcomes were favorable. Formenti et al.^13^ reported on 98 patients after a median follow‐up of 64 months. Cosmesis was rated good/excellent in 89% of patients, and there were only two grade 3 toxicities. William Beaumont hospital reported on 94 patients who were treated with an accelerated partial breast regimen planned with three‐dimensional radiotherapy.^16^ After a 48‐month follow‐up, they reported a 4% grade 3 toxicity and 89% good/excellent physician‐assessed cosmesis. Both reports had similar mean PTV/ipsilateral breast volume ratios, 18% and 17% for Formenti and Beaumont hospital, respectively, and were approximately 50% larger than this report. Mean PTV values were 200.8 and 268.1 cm^3^, respectively.

Hepel and K Leonard et al. reported separate results in 2010 and 2012, respectively, from Tufts University.^20^, ^38^ Both of these publications reported an undesirably high rate of unfavorable outcomes and confirmed their relationship to dose‐volume parameters. In short, these reports found that increasing PTV/whole‐breast volume ratios, isodose volumes, and planning target volumes correlated with clinical outcomes such as fibrosis and undesirable cosmesis.

The Canadian RAPID trial recently has been published^39^, ^40^ and their results show that at 3 years there was a significantly higher probability of adverse cosmesis in the APBI cohort than the whole‐breast radiotherapy group (29% vs. 17% *p* < 0.001 as judged by nurses and 26% vs. 18% *p* = 0.0022 as judged by patients). Upon a multivariate analysis, they also reported that tumor location, a current smoking status, seroma volume, and older age were significantly associated with an adverse cosmesis at 3 years. Their trial was specified as 3D‐CRT or IMRT however both papers explain that either 3D‐CRT or IMRT planning was allowed and does not state what proportion of patients were treated with either technique. Also, although dosimetry planning constraints were stated, there were no stated statistics which addressed what the actual planning volumes were in any of the patient cohorts. In their APBI patients, both seroma and breast volumes were found to be a significant variable in an adverse cosmesis at 3 years.

Results of our study demonstrate that patient selection, as well as strict dosimetric constraints, are very important to achieve optimal results in accelerated partial breast radiation. Regardless of whether IMRT or 3D‐CRT is used for delivery, patients generally experience less breast pain over time, as they recover from local therapy. However, IMRT resulted in significantly more pain resolution at 2 years than 3D‐CRT. There was also a trend for pain improvement (*p* = 0.058) at 3 years, which may become significant with longer follow‐up as fewer patients were available for analysis at 3 years. One potential explanation for the lower amounts of long‐term pain with IMRT likely relates to the greater conformality of this technique. Our results demonstrate that the CI_100_ correlated with the maximum pain reported by patients. There was also a correlation between the CI_100_ <0.37 and maximum patient‐reported pain. In our analysis, IMRT was able to achieve a mean CI of 0.31, whereas this was 0.44 for 3D‐CRT. Greater conformality of the higher radiation doses likely leads to less fibrosis and/or edema, both of which can contribute to pain and discomfort. Since these are typically chronic toxicities of radiation, the differences in pain resolution seen in our study were not appreciated until the 2‐year time point. Further follow‐up is necessary to determine if this pain improvement with IMRT persists to 3 years and beyond.

In conclusion, since many early stage breast cancer patients will have prolonged survival times, it is critical that adjuvant therapies not only ensure lower rates of recurrence but also attempt to minimize long‐term toxicities that may impact the quality of life. Since the intent of breast conservation therapy is to preserve and maintain the integrity of the unaffected breast tissue, the optimal radiation regimen should further support that intent and protect as much normal tissue as possible which should lead to lower risks of long‐term pain and altered cosmesis. A degree of caution has to be understood concerning our findings due to several limitations. First, there was no independent and/or blinded evaluation of MD‐related cosmesis. In addition, our analysis did not include any potential role that chemotherapy may have had in cosmetic outcome or beast pain. As well, there were only 72 and 75 patients in the 3D‐CRT and IMRT cohorts, respectively, at 60 months. This latter point may weaken any final analysis of cosmesis at that 60‐month timeframe especially since in years 1–4 there were no significant differences between the two cohorts. Therefore, drawing definitive conclusions concerning cosmesis in our patient population is problematic. Additional caveats of IMRT also include a higher integral dose of a lower dose exposure to a larger area, a higher treatment machine monitor unit output and, as well it has a higher expense.

Nonetheless, according to the findings of our study, we continue to believe IMRT may be a better delivery technique for APBI based on the aforementioned improvements in patient‐reported pain. These findings support moving forward with IMRT as also described in the Florence trial which compared every other day IMRT APBI to a whole‐breast tangential technique.^41^ This trial has further defined treatment practice by establishing this once every other day treatment as a viable alternative to the BID fractionation described in this report. We continue to advocate for IMRT in appropriately selected early stage breast cancer patients, according to our inclusion criteria of this trial. Future efforts will include maintaining long‐term follow‐up of our patients to see if our significant findings persist, establishing possible dosimetric parameters for the conformality indices, and advocating for third‐party payers to include IMRT as an acceptable approach for APBI.

## CONFLICT OF INTEREST

None.

## ETHICS STATEMENT

All patients were enrolled/consented for one of two protocols approved by WIRB(initial approval 7/7/09)‐20091193; Clinical Trials gov ID NCT01185132 and WIRB(initial approval 1/30/04)‐20040075; Clincial Trials gov ID NCT01185145.

## Data Availability

All patient data are private and reposed at Rocky Mountain Cancer Centers in both digital data and paper/EMR chart. Upon request, non‐patient identifiable data can be provided.
